# Effect of different bulking agents on the quality, microbial community structure and metabolic functions during human feces composting in foam composting device

**DOI:** 10.3389/fmicb.2025.1556537

**Published:** 2025-06-10

**Authors:** Tianyang Ning, Xiangqun Zheng, Jiayin Liang, Weihan Wang, Guowei Zhang, Xiaocheng Wei, Lu Tan

**Affiliations:** ^1^Agro-Environmental Protection Institute, Ministry of Agriculture and Rural Affairs, Tianjin, China; ^2^Key Laboratory of Rural Toilet and Sewage Treatment Technology, Ministry of Agriculture and Rural Affairs, Tianjin, China; ^3^Tianjin Municipal Engineering Design & Research Institute Co., Ltd., Tianjin, China

**Keywords:** human feces, bulking agent, compost quality, microbial community structure, metabolic functions

## Abstract

Aerobic composting represents an efficacious strategy for the disposal of human feces, yet investigations into the effects of different bulking agents on this process remain limited. This study investigated the effects of composting human feces with four types of bulking agents—wheat straw, corn straw, millet straw, and sawdust—in a foam composting device, as well as the impacts of the process on the microbial community structure and metabolic functions adopting sequencing data analysis and metagenomic analysis. The results demonstrate that aerobic composting can safely treat human feces, resulting in a mature compost product. Comparative assessments of compost quality and microbial profiles with various bulking agents indicated superior performance of corn straw compost, surpassing those produced with wheat straw, millet straw, and sawdust in terms of humification level (HA/FA = 2.9), peak temperature reached (71.2°C), composting duration (20 days), and nutrient composition (TN 42.87 g/kg). Additionally, the diversity and dominance of certain microbial colonies (Firmicutes, Actinobacteria, Proteobacteria, and Bacteroidota) were significantly higher in composts formulated with corn straw. The metagenomic data analysis reveals significant differences in the abundance of “carbon metabolism” and “microbial metabolism” among different groups, further indicating that the addition of different bulking agents affects the utilization of metabolic products, amino acids, and carbohydrates as carbon sources by microbes in human feces compost. Consequently, leveraging corn straw as a bulking agent, given its abundant availability, could potentially improve the efficiency and outcome of the human feces composting process.

## 1 Introduction

Achieving universal access to secure sanitation facilities is pivotal for the United Nations Sustainable Development Goals (SDGs), bearing significant impact. United Nations projections indicate an escalation of the global population to approximately 8.5 billion by 2030 and 9.7 billion by 2050 (Bossone et al., 2020), this growth rate directly leads to a rapid increase in the total amount of human excrement. It is estimated that by 2030, human fecal waste will reach around 1.0 × 10^12^ kg annually (Berendes et al., 2018), with over half (57%) of this quantity unprocessable by centralized sewerage systems (Cheng et al., 2022), this means that over 570 billion kilograms of human feces will face the dilemma of ineffective treatment every year. Currently, about 4.5 billion individuals lack access to adequately managed sanitation services (McNicol et al., 2020). Therefore, it is crucial to develop affordable, easy to operate, and safe and reliable sanitation treatment solutions for the massive production of human feces. This links population growth with the amount of human feces produced and the urgent need for human feces treatment.

Human feces are rich in essential nutrients (nitrogen, phosphorus, potassium) and organic matter. Resource recovery from fecal matter has emerged as a viable approach to enhance the functional and economic value of sanitation systems, beyond their primary roles in reducing environmental pollution and health risks (Harder et al., 2019; Li D. et al., 2023; Trimmer et al., 2019). International organizations such as the World Health Organization (2006) have released documents such as Guidelines for the Safe Use of Wastewater, Excreta and Greywater, provided support for the resource utilization and composting of human feces. In parallel, China has established a regulatory system through national standards and technical specifications such as GB/T 7959-2012 (Hygienic Requirements for Excreta Treatment) and the Guidelines for Harmless Treatment and Resource Utilization of Rural Toilet Manure issued by the Ministry of Agriculture and Rural Affairs of the People’s Republic of China, which collectively standardize practices in feces waste management and agricultural recycling within the national context. Composting toilets, a type of water-free, environmentally friendly, cost-effective, and low-maintenance sanitation system, utilize aerobic processes to treat human waste, converting it into humus-like, nitrogen-rich material. This nutrient-rich compost can be repurposed locally as high-quality organic fertilizer and soil conditioner (Gao et al., 2017; Lam et al., 2022; Wang et al., 2015; Zhou et al., 2018). Suitable for rural and water-scarce areas, composting toilets are increasingly adopted in institutional and suburban settings as well. While composting toilets offer a promising solution, their operational efficiency depends heavily on optimizing the composting process itself—a challenge that necessitates further scientific investigation.

The composting of organic matter in manure is intrinsically slow, attributed to its low carbon-to-nitrogen (C/N) ratio of approximately 10 and elevated moisture content (Meng et al., 2021). To expedite this process, the incorporation of high-carbon bulking agents like straw is indispensable for achieving an optimal C/N ratio of 20–30 and for moisture reduction. The choice of bulking agents significantly influences compost quality, maturation time, and its effectiveness as a harmless soil amendment. For instance, when used in pig manure composting within a rotary drum system, sawdust outperforms dry leaves and straw in enhancing thermal resistance and improving the quality of the final compost product (Lalthlansanga et al., 2023). Furthermore, composts made from mushroom waste combined with straw and pig manure exhibit superior quality over those using sawdust and rice husk mixtures. Regarding nitrogen conservation during human feces composting, sawdust serves as a superior substrate compared to coffee husks and brewery waste (Manga et al., 2022). These findings highlight the critical role of bulking agents selection in shaping both the physicochemical and biological dynamics of composting systems. In conditions with an initial carbon-nitrogen (C/N) ratio of 20:1, the application of distiller’s grains as an additive in pig manure composting not only bolsters microbial community diversity but also enhances the immobilization of heavy metals like Cu and Zn, outperforming maize straw and green waste in this respect (Guo et al., 2022). The selection of bulking agents critically determines the duration needed for compost to achieve stable organic matter content and influences the biochemical decomposition pathways of organic materials (Doublet et al., 2011). Employing coffee husks as a substrate for composting human fecal sludge has been shown to elevate pathogen deactivation rates, thereby reducing the composting period from 8 weeks, as observed with sawdust and brewery waste, to 6 weeks (Manga et al., 2021). The benefits of using sawdust as a substrate for chicken manure composting significantly surpass those of mushroom residue regarding composting efficiency and cost-effectiveness (Liu S. et al., 2024). Research indicates that composting with corn straw is more effective in lignin degradation compared to wheat straw (Zhang et al., 2016). Furthermore, compost produced from straw combined with chicken manure demonstrates superior lignin degradation and humic acid formation compared to that made from straw and pig manure (Zhao B. et al., 2022). Despite these advances, the majority of existing studies have focused on animal manure, leaving a critical gap in understanding human feces composting processes.

Although numerous studies have compared the effects of various bulking agents on compost, most have focused on animal manure, with limited research on human feces composting. Human feces treated through lactic acid fermentation combined with thermophilic composting exhibit greater sanitation and fertilizer efficiency than when paired with vermi-composting (Andreev et al., 2017). Under identical conditions, human feces compost shows lower maturity, humification, and quality compared to cow manure compost (Li D. et al., 2023). Based on compost maturity NI and other indicators, the optimal mixing ratios for human feces, pig manure, and straw compost were determined to be 13.7%, 41.4%, and 44.9%, respectively, through experimental and model comparisons (Gao Y. et al., 2021; Gao et al., 2022). Composting human feces with green cuttings and straw significantly reduces pathogens and ARGs within the pile (Werner et al., 2022). Thermophilic composting is an effective method to maintain high organic nitrogen levels in human feces compost (Bai and Wang, 2010). However, the variability in regional agricultural waste composition raises unresolved questions about the universal applicability of these materials for human feces composting. This necessitates further research to evaluate the compatibility of region-specific bulking agents with human fecal matter under diverse environmental and operational conditions.

In this study, we established four experimental groups utilizing different types of bulking agents for composting: corn straw, wheat straw, millet straw, and sawdust, all mixed with human feces. These four bulking agents were selected based on the varying types of agricultural waste available in different regions. The primary objective is to investigate the effects of these bulking agents on the quality and maturity of human feces composting. Additionally, we aim to elucidate how different bulking agents influence the diversity, composition, and function of compost microorganisms, as well as their relationships with physicochemical factors and functional compost maturity. This research will enhance our understanding of the human feces composting process and provide guidance for selecting appropriate bulking agents for this purpose.

## 2 Materials and methods

### 2.1 Experimental materials and setup

The experiments were carried out at Agro-Environmental Protection Institute located in Nankai District, Tianjin, China. Raw materials include human feces (HF), corn straw (CS), millet straw (MS), wheat straw (WS), and sawdust (SD). Their physicochemical properties including total carbon (TC), total nitrogen (TN), cellulose, hemicellulose, and lignin are provided in Table 1. Human feces were collected from a rural pit toilet in Tianjin, which was a yellowish, mud-like substance. The crop straws (CS, MS, WS) and sawdust (SD) were procured from the Livestock and Poultry Manure Resource Utilization Center in Yangjiapo Town, Tianjin, and were pulverized to a granularity of 2–5 cm. Separate HF from CS MS, WS, and SD were mixed in wet weight ratios of 2.5/1, 2.5/1, 2.5/1, and 5/1 to achieve a C/N ratio of around 20. The moisture content and C/N ratio of human feces and four types of bulk agents are shown in Supplementary Table S1. Their moisture content was adjusted to 60%–70% using distilled water. A microbial agent designed for feces composting, from Hebi DaDe Biological Science and Technology Co., Ltd., was added at a ratio of 1:10,000 of the dry weight. The experimental setup was then loaded into the foam boxes, fill the compost material to 2/3 of the box. The foam box used is 800 mm long, 400 mm wide, 475 mm high and 30 mm thick. The material is polystyrene (EPS) foam, lightweight and low cost. The foam material has good heat insulation performance, can maintain the temperature in the composting process, and promote microbial activity.

**Table 1:** Main component of bulking agents and human feces.

Raw material	TC (g/kg)	TN (g/kg)	Cellulose (g/kg)	Hemicellulose (g/kg)	Lignin (g/kg)	Lignocellulose (g/kg)
HF	498.71	40.70	82.05	0	191.12	273.34
CS	471.12	9.78	269.15	146.01	185.23	600.11
MS	467.61	8.69	268.22	113.12	179.31	560.08
WS	442.10	7.34	259.32	155.14	191.09	605.24
SD	513.83	3.40	376.15	174.11	242.04	792.31

### 2.2 Composting process and sample collection

Composting ingredients are thoroughly turned every 2 days. Samples were collected from both composting groups on days 0, 3, 6, 9, 12, 15, 18, 21, 25, and 31. This sampling method ensures that samples are taken in all four stages of composting: mesophilic Phase, thermophilic Phase, cooling Phase, and maturation Phase. About 200 g samples were collected at a time according to the “five-point sampling method” and mixed nicely. The samples were divided into three aliquots, of which one was directly measured for pH and EC, one was air-dried for TN and total phosphorus (TP) measurement, and the remaining one was stored at −80°C for microbiological analysis.

### 2.3 Determination of physicochemical properties

Compost temperature was recorded daily at 4:00 pm using a digital temperature probe (Hengshui Zhengxu Electronic Technology Co., Ltd. LCD-105461). Samples were mixed with deionized water at a 1:5 ratio, shaken for 1 h, and the pH and electrical conductivity (EC) of the suspension were measured after 24 h of equilibration. The pH and EC of compost were measured using Thermo Scientific Orion Star A221 and Thermo Scientific Orion Star A325. Total nitrogen (TN) and total phosphorus (TP) concentrations were determined using hydrazine reduction and molybdenum blue methods, respectively, with a continuous flow autoanalyzer (AA3, Seal Analytical, Germany), following the peroxydisulfate oxidation process as outlined in our previous study (Tan et al., 2019).

For humic acid extraction, 1 g of the sample was treated with 10 mL of a 0.1 M Na_4_P_2_O_7_·10H_2_O and NaOH solution. The mixture was agitated for 24 h at 25°C, then centrifuged at 11,000 rpm for 15 min, and filtered through a 0.45 μm Millipore membrane. Fulvic acids were isolated by adjusting the pH of the humic acid solution to 1.5 using 6 M HCl, followed by a 12-h incubation at 4°C and centrifugation at 11,000 rpm for 15 min. The supernatant was collected as fulvic acid, while the precipitate was identified as humic acid. The humic acid was washed twice with alternating 0.1 M HCl and deionized water, then dissolved in 0.05 M NaHCO_3_ (Wu et al., 2020). Concentrations of humic and fulvic acids were measured using a Shimadzu TOC-Vcph analyzer. Each procedure was performed in triplicate, and the mean value was calculated.

### 2.4 DNA extraction and metagenomic sequencing

Sample DNA was extracted utilizing the E.Z.N.A.® Soil DNA Kit (Omega Bio-tek, United States). Post-extraction, DNA concentration and purity were quantified, while DNA integrity was verified via 1% agarose gel electrophoresis. The DNA was then fragmented to an approximate size of 400 bp using a Covaris M220 (Gene Company Limited, China), followed by the construction of a paired-end library using the NEXTFLEX Rapid DNA-Seq kit (Bioo Scientific, Austin, TX, United States). Paired-end sequencing was conducted on an Illumina NovaSeq 6,000 platform (Illumina Inc., San Diego, CA, United States) at Majorbio Bio-Pharm Technology Co., Ltd. (Shanghai, China), in accordance with the NovaSeq Reagent Kits’ manufacturer protocols.^1^

### 2.5 Taxonomic and functional gene annotation

Sequencing data analysis and metagenomic analysis were conducted using the Majorbio Cloud Platform, a free online service.^2^ Illumina paired-end reads were first subjected to adaptor trimming and low-quality read removal utilizing fastp (Chen et al., 2018).^3^ Subsequently, metagenomic assembly was performed with MEGAHIT (Li et al., 2015),^4^ where contigs of at least 300 bp were selected for subsequent gene prediction and annotation processes. Open reading frames (ORFs) of each assembled contig were predicted using Prodigal (Hyatt et al., 2010) or MetaGene (Noguchi et al., 2006).^5^ A non-redundant gene catalog was then constructed via CD-HIT (Fu et al., 2012)^6^ with criteria of 90% sequence identity and 90% coverage. Gene abundance was calculated by aligning high-quality reads to the non-redundant gene catalog using SOAPaligner (Li et al., 2008).^7^ Representative sequences were aligned to the NR database for taxonomic annotation using Diamond (Buchfink et al., 2015)^8^ with an e-value threshold of 1e^−5^. KEGG annotation was performed by aligning against the Kyoto Encyclopedia of Genes and Genomes database^9^ using Diamond (Buchfink et al., 2015) (version 0.8.35) with the same e-value cutoff of 1e^−5^.

### 2.6 Data analysis

The line graphs representing the temporal variations in composting temperature and physicochemical properties were drawn using Origin software version 2021 (Origin Lab Corp., Northampton, MA, United States). Box plots were generated using the Majorbio cloud platform to show the differences in microbial diversity between groups, and the significance was tested using the Kruskal-Wallis H test. Heatmaps were generated using the Majorbio cloud platform to display the relative abundance of microbial phyla and genera in compost, and bar graphs were plotted to show the species differences among microbial communities at the phylum and genus levels in each group, identifying significantly different microbes, the significance was tested using Kruskal-Wallis H test. Network analysis was conducted using Gephi 0.9.2 based on Spearman’s correlation coefficient (*p* < 0.05). Mental-Test heatmaps were generated on the Majorbio cloud platform to assess the impact of physicochemical properties on microbial and functional gene composition. Partial least squares path modeling was performed using SmartPLS3.0.

## 3 Results and discussion

### 3.1 Physicochemical properties of the composts

#### 3.1.1 Humification degree

**Figure 1:**
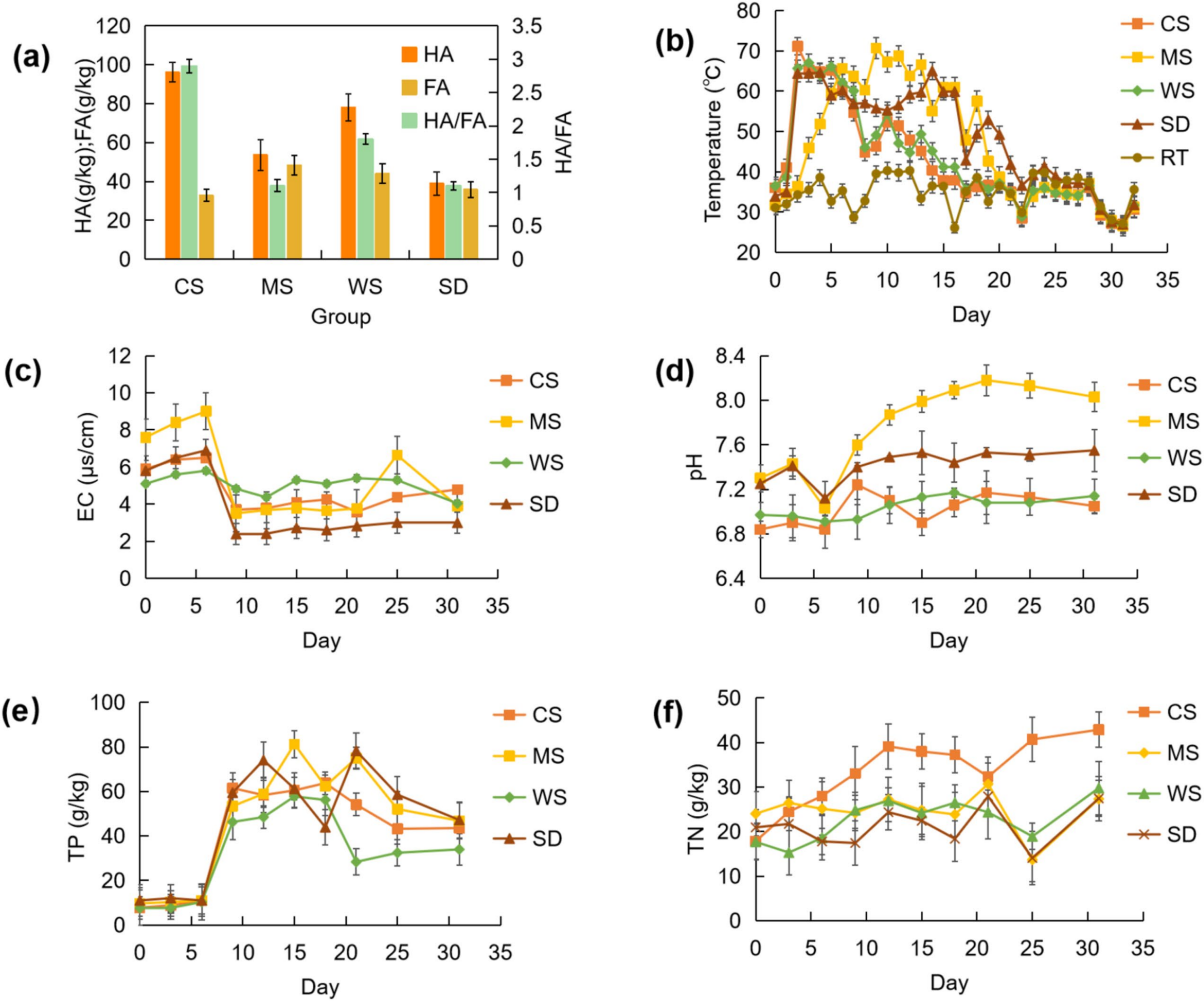
Variation of HA & FA **(a)**, temperature **(b)**, EC **(c)**, pH **(d)**, TP **(e)**, and TN **(f)** during composting.

Humic substances (HS) serve as critical compost byproducts, enhancing soil pH stability, water holding capacity, and nutrient dynamics (Abdellah et al., 2022; Guo et al., 2019; Yang et al., 2024). The primary constituents of HS are humic acids (HA) and fulvic acids (FA) (Chen et al., 2023). Our observations indicated HA concentrations in human feces composts ranged from 39.0 to 96.2 g/kg, with residual FA levels reaching 32.8 to 48.4 g/kg at compost maturity (see Figure 1a). The microbial processes can convert HA and FA, with increased HA content signifying enhanced compost maturity and quality due to HA’s higher molecular weight and greater stability compared to FA (Zhou et al., 2014). Consequently, the HA to FA ratio (HA/FA) has become a reliable indicator of compost humification and maturity (Li et al., 2017). Among the examined groups, the CS group exhibited the highest HA/FA ratio at 2.9, surpassing the MS (1.1), WS (1.8), and SD (1.1) groups. Notably, the low HA/FA ratios observed in the MS and SD groups align with recent findings that suggest human feces compost concludes with an HA/FA ratio of 1.05, indicative of less humification compared to cow manure composting (Cao et al., 2021; Li D. et al., 2023). Remarkably, the HA/FA ratio of the CS group reached 2.9, exceeding those reported for cow (1.34~1.78), chicken (1.9~2.6), and pig (0.869~1.67) manure composts in recent studies (Shan et al., 2024; Su et al., 2024; Wang et al., 2024), highlighting the potential for achieving substantial humification and maturity in human feces compost with suitable bulking agents.

#### 3.1.2 Temperature change and compost stages

Temperature serves as a robust metric for monitoring the composting process (Li et al., 2023b). Significant temperature disparities were observed across the four composting regimens. As delineated in Figure 1b, the CS group exhibited the highest composting temperatures and the shortest composting duration relative to the other groups. Each treatment group transitioned into the mesophilic phase (>35°C) on day one, advancing to the thermophilic phase (>50°C) by day three (Figure 1b). The apex temperature attainment varied; the CS group peaked on day two at 71.2°C, followed by the MS group on day nine (70.7°C), the WS group on day three (67.1°C), and the SD group on day 14 (65°C). The thermophilic phase persisted for 6 days in both the CS and WS groups, 16 days in the MS group, and 15 days in the SD group. All groups satisfied the parameters for generating non-hazardous compost, with thermophilic phases exceeding 3 days, a duration deemed sufficient for nullifying pathogenic microorganisms (Yang and Zhang, 2022). The protracted thermophilic interval in the SD group likely reflects delayed lignin decomposition (Meng X. et al., 2019), whereas the extended high-temperature period in the MS group could be attributed to reduced oxygen levels within its compost, as studies indicate longer elevated temperature durations in composts with diminished aeration (Gao et al., 2010). Composts comprised of corn and wheat straw reverted to ambient conditions by day 16, notably 5–7 days earlier than those of the MS and SD groups, indicating rapid maturation of human feces compost with corn straw. Higher temperatures (such as the thermophilic stage above 50°C) can promote the decomposition of organic matter by microorganisms, affect the transformation of carbon, nitrogen and other elements in compost, and promote the process of humification. Studies have shown that faster heating rates and higher temperatures increase the degradation rates of cellulose, hemicellulose, and lignin by 78%, 10%, and 109%, respectively, compared to traditional composting, while promoting humification processes (Zhu et al., 2021). In this study, the CS group had the fastest heating rate and the highest composting temperature, and also had the highest HI index (HA/FA) at the end of composting, which is consistent with the conclusions of the above studies.

#### 3.1.3 Changes of EC

Electrical conductivity (EC) serves as an indicator of compost suitability for plant growth (Yang et al., 2020). As shown in Figure 1c, the EC of all treatments initially increased (from 5.1–7.6 to 5.8–9), then decreased, and eventually stabilized at the end of composting (3–4.79). By the end, the EC values ranked as follows: CS (4.79) > WS (4.04) > MS (3.9) > SD (3). The initial rise in EC across all groups may result from moisture evaporation and partial organic matter loss during the pile heating phase, consistent with previous findings (Villa Gomez et al., 2019). The subsequent decrease in EC likely correlates with the rapid growth of aerobic microorganisms, reducing NH_4_^+^ content in the pile (Kalamdhad and Kazmi, 2009). The extended thermophilic phases in the MS and SD groups, possibly linked to increased microbial activity, may explain the more pronounced EC reduction observed in these treatments.

#### 3.1.4 Change of pH

The pH plays a crucial role in composting efficiency (Wang S.-P. et al., 2017). As shown in Figure 1d, the pH of all groups exhibited a general upward trend with minor fluctuations, increasing on average from 7.09 to 7.44. The MS group (7.3–8.03) showed a more significant pH rise compared to the other groups (6.84–7.05, 6.97–7.14, and 7.25–7.55). Previous studies indicate that the optimal pH range for composting is 6.7–9.0, within which microbial activity is maximized (Meng et al., 2020). The pH of compost can affect the degradation of lignocellulose and the formation of humus by microorganisms. The treatment of adjusting pH and inoculating with P. chrysosporium resulted in a significant decrease in lignin content and a significant increase in humic acid content at the end of composting (Zhao et al., 2023). During the composting process in this study, the pH remained within an appropriate range, and all treatment groups reached maturity after composting was completed. The pH of the MS and SD groups declined during the thermophilic phase, likely due to factors such as smaller particle size, higher moisture content, compact pile structure, and localized acidification. As rotation frequency increased, organic acids decomposed, and the pH rose due to ammonia formation. The sharp pH rise observed in the CS group on day 10 may result from a slight temperature increase on days 9 and 10, which could have accelerated microbial reproduction and ammonia production.

#### 3.1.5 Changes of nutrient content

Nutrient content is a key parameter for evaluating compost quality. As shown in Figure 1e, the initial total nitrogen (TN) content ranged from 17.69 to 24 g/kg, with the WS group having the lowest (17.69 g/kg) and the MS group the highest (24 g/kg). Post-composting, TN content increased across all groups, with the CS group showing the largest rise (140.44%). This increase in TN is likely associated with organic matter decomposition and nitrogen fixation, which was particularly elevated in the CS group. Alkaline conditions (pH > 7) promote the volatilization of NH_3_, leading to a decrease in TN; Acidic conditions (pH < 6) inhibit ammonification and reduce nitrogen loss (Zhao et al., 2023). The pH of the CS group remained consistently low, with the highest TN concentration at the end of composting. Figure 1f illustrates that total phosphorus (TP) in all groups followed an upward trend with fluctuations throughout composting. By the end of the process, TP levels had risen by 456.9%, 382.5%, 344.7%, and 327.2% in the CS, MS, WS, and SD groups, respectively, with the CS group showing significant phosphorus accumulation. This “concentration effect” (Wei et al., 2022) arises because organic matter is lost as water, CO₂, NH_₃_, and other volatile compounds, whereas phosphorus, being less susceptible to transformation, remains in the pile, leading to a continuous increase in TP across all groups.

### 3.2 Changes in microbial community

#### 3.2.1 Microbial diversity

**Figure 2:**
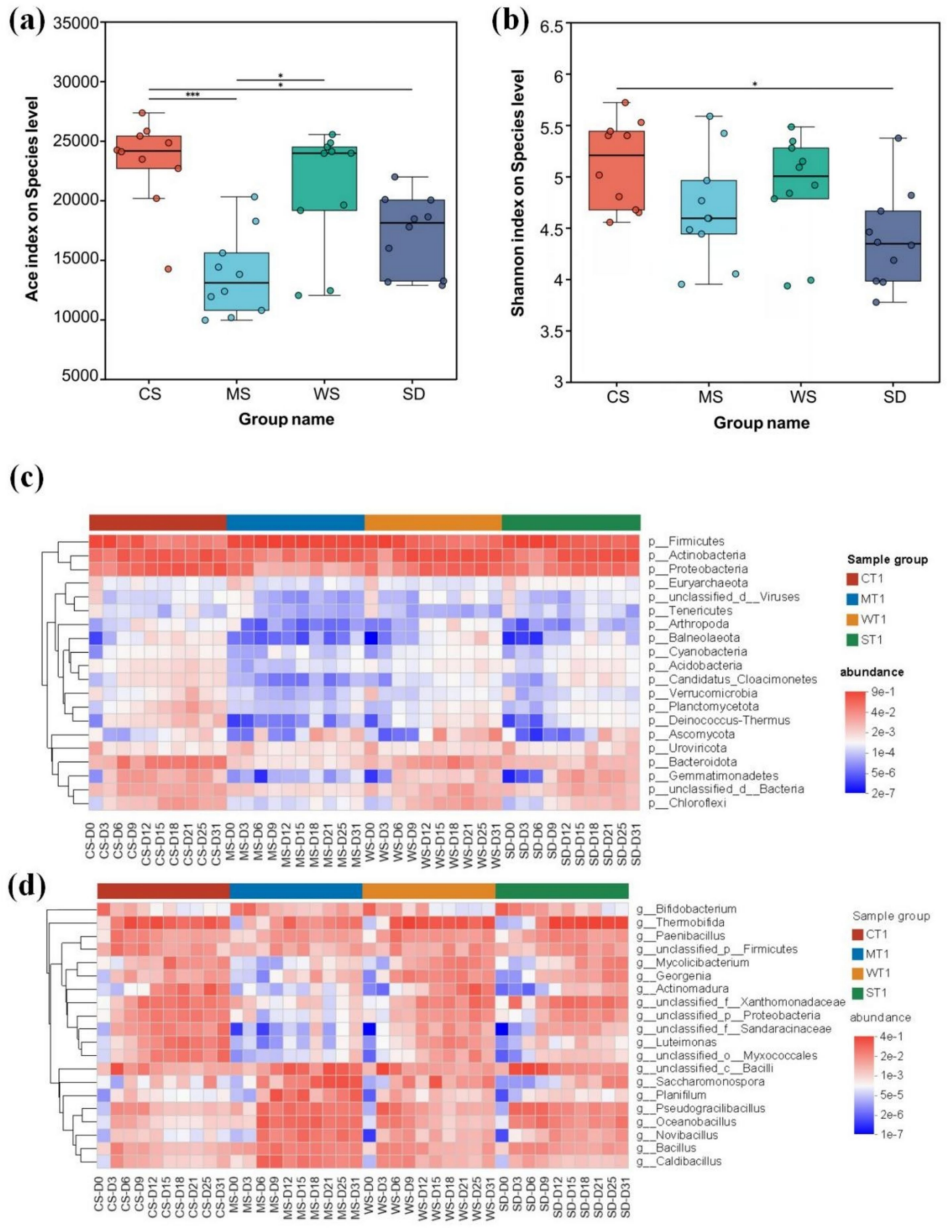
**(a)** Ace and **(b)** Shannon index of microorganisms in different bulking agents, heat maps of microbial **(c)** phyla and **(d)** genus levels in different bulking agents groups. The color of the elements in the heatmap is determined by the relative abundance of the corresponding microorganisms.

Microorganisms drive the biochemical reactions in composting, with microbial community composition and succession playing a crucial role in substrate transformation (Duan et al., 2022). In this study, significant variations in microbial diversity were observed across different composting bulking agents, as indicated by the Ace and Shannon indices. As shown in Figure 2a, the CS group exhibited the highest Ace index, followed by WS. The Ace index of CS was significantly higher than that of MS (*p* < 0.001) and SD (*p* < 0.05). A similar pattern was noted in the Shannon index, with microbial diversity ranked as CS > WS > MS > SD. These findings suggest that CS compost harbors a more diverse microbial community.

#### 3.2.2 Microbial community structure

As indicated by the PCoA analysis, distinct differences in microbial community structure were observed across various composting stages and bulking agents (Supplementary Figure S1). Specifically, the dominant phyla in human feces compost included *Firmicutes* (5.0~92.33%), *Actinobacteria* (1.99~60.13%), *Proteobacteria* (0.76 ~ 47.91%), and *Bacteroidota* (0.08~13.98%) (Figure 2c), aligning with previous findings on kitchen waste and pig manure composts (Gao X. et al., 2021; Zhu et al., 2020). Among these, *Firmicutes* exhibited a marked increase during the thermophilic phase, likely due to their ability to form heat-resistant endospores, conferring a competitive advantage in high-temperature conditions (Zhong et al., 2018; Li et al., 2023b). The MS group also showed a significantly higher abundance of *Firmicutes* (*p* < 0.001, Supplementary Figure S2a), possibly due to its extended thermophilic period. *Actinobacteria*, key contributors to organic matter decomposition and humification (Wei et al., 2019), continued to increase across all groups throughout composting. *Proteobacteria* and *Bacteroidota* were enriched in the final composting stage, a trend commonly observed in other composting processes (Wei et al., 2018). *Proteobacteria* becomes the dominant bacterium after a decrease in temperature, promoting humification and nutrient fixation (Zhong et al., 2018). However, their abundance was lower in the MS group (*p* < 0.001, Supplementary Figure S2a), likely due to reduced adaptation to the extreme temperatures and prolonged thermophilic phase characteristic of this group (Zhao et al., 2016). Additionally, the relative abundance of *Gemmatimonadetes* and *Chloroflexi* increased after composting, consistent with reports on animal manure and food waste composts (Peng et al., 2023; Wang et al., 2022; Zhong et al., 2018).

At the genus level, bacteria such as *Thermobifida* (0.002%~47.06%), *Caldibacillus* (0.003%~13.73%), *Pseudogracilibacillus* (0.002%~15.39%), *Oceanobacillus* (0.01%~17.33%), *Actinomadura* (0.00003%~20.33%), and *Bacillus* (0.09%~7.58%) were abundant and enriched during the thermophilic and mesophilic phases of composting (Figure 2d). *Bacillus* is active at high temperatures and is responsible for decomposing complex organic compounds such as cellulose and hemicellulose. Higher temperatures can increase the abundance of *Bacillus*, thereby increasing the decomposition rate of organic compounds (Zhu et al., 2021). At the same time, during the composting process, the pH remained between 6 and 9, and *Bacillus* was active, promoting the breakdown of protein and fat (Wan et al., 2020). These genera are commonly observed in composting processes and are primarily associated with organic matter transformation and the breakdown of cellulose and lignocellulosic compounds (Anderson et al., 2011; de Gannes et al., 2013; Dias-Neto et al., 2013; Wushke et al., 2015; Zhang et al., 2015). *Bifidobacterium*, a typical commensal bacterium in the human gut (Kose et al., 2018), gradually decreased throughout the composting process. During the high temperature period, thermophilic bacteria dominate the decomposition of organic matter, while during the medium temperature period, humifying microorganisms take over, forming a phased functional division of labor (Wang et al., 2022). At the same time, acidic conditions promote fungal decomposition of lignin, while bacteria dominate nitrogen cycling under neutral to alkaline conditions, and high C/N ratios supplement nitrogen sources through nitrogen fixing bacteria (Anderson et al., 2011).

The Kruskal-Wallis H test revealed no significant differences in the abundance of *Saccharomonospora*, *Oceanobacillus*, *Bacillus*, *Planiflum*, and *Novibacillus* across the CS, WS, and SD groups. In contrast, their abundance in the MS group was significantly higher (Supplementary Figure S2b). These microorganisms have been detected or isolated from compost piles during thermophilic or mature stages (Chen et al., 2022; Duan et al., 2023; Yu et al., 2021). *Saccharomonospora*, a member of *Actinobacteria*, thrives in thermal environments and is capable of converting lignin and cellulose into humic substances (Shivlata and Satyanarayana, 2015; Wang et al., 2019). The presence of *Oceanobacillus* in the CS, WS, and SD groups was merely 4.07%, 9.68%, and 39.53%, respectively, of its abundance in the MS group. Similarly, *Planiflum*’s abundance in the CS, WS, and SD groups constituted just 1.19%, 1.19%, and 1.03%, respectively, of that in the MS group, highlighting its capacity for synergistic degradation of hemicellulose, cellulose, and macromolecular proteins (Zhang et al., 2021). Consequently, these findings suggest the elevated temperatures and extended duration of the thermophilic stage in the MS group promote the growth of these thermophilic microorganisms.

### 3.3 Co-occurrence network and keystone genera

**Figure 3:**
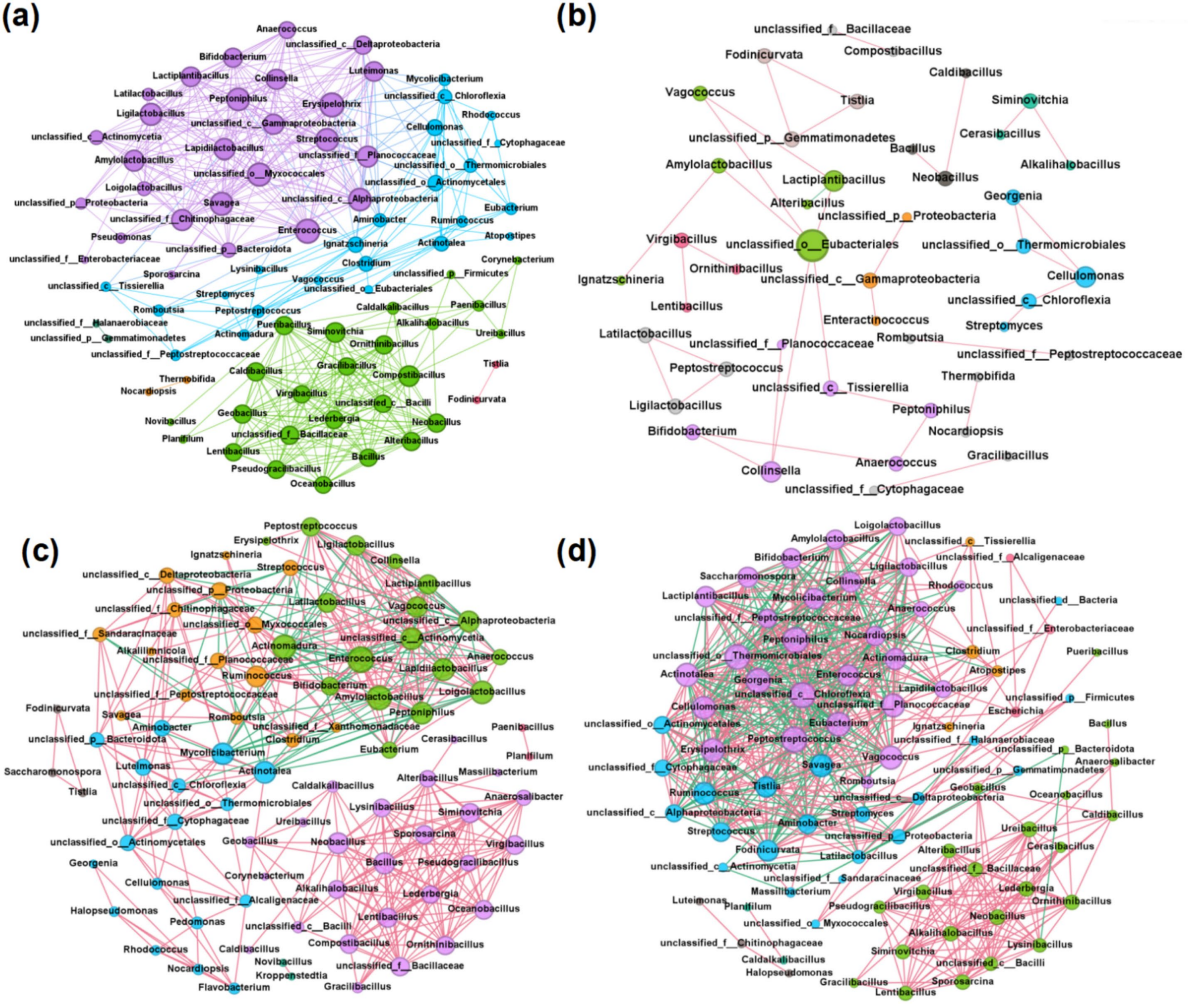
Microbial network diagrams for different bulking agents, including **(a)** CS group, **(b)** MS group, **(c)** WS group, and **(d)** SD group. Nodes represent microbes, and edges represent relationships between microbes. The color of the nodes is distinguished by modularity. Node size was differentiated by degree, and edge color was distinguished by correlation, with pink representing positive correlation and green indicating negative correlation.

Network analysis can enhance our understanding of the stability and functionality of compost microbial community structures (Liu et al., 2023). To discern the co-occurrence patterns among microbes and the impact of different bulking agents on microbial communities, a network analysis was performed involving the top 100 genera, selected based on their significant correlations (r > 0.8, *p* < 0.01). According to Table 2, the network topologies of the CS, WS, and SD groups exhibit higher average degrees, graph densities, and clustering coefficients compared to the MS group, suggesting more intense microbial interactions within these groups (Deng et al., 2012; Zhao Y. et al., 2022). According to Figure 3, the MS group’s network displayed the fewest total edges, indicative of the lowest complexity and potentially inferior microbial stability and environmental resilience (Yang et al., 2019). Furthermore, each of the four networks featured a predominance of positive over negative edges, highlighting a greater prevalence of cooperative symbioses rather than competitive interactions among microbes throughout the composting process (Faust and Raes, 2012).

Keystone genera in human feces compost were identified through analysis of network topological features, primarily comprising *Proteobacteria*, *Firmicute*s, *Actinobacteria*, and *Chloroflexi*. Notably, overlaps in keystone genera were observed across different groups. For example, *Peptoniphilus* and *Enterococcus* were identified as keystone genera in both the CS and WS groups, with *Peptoniphilus* also serving as a keystone genus in the SD group. These genera are significant components of human commensal flora and are known opportunistic pathogens linked to bloodstream, soft tissue, joint, and surgical site infections (Qian et al., 2021). Post-composting, their average abundance markedly decreased from 3.8% to 0.0046% and from 2.7% to 0.034%, respectively, indicating a reduction in fecal pathogens. Additionally, lactic acid bacteria (LAB) such as *Ligilactobacillus*, *Lapidilactobacillus*, and *Lactiplantibacillus* were identified as another group of keystone taxa. Numerous studies have demonstrated that LAB, when used as microbial promoters, can accelerate the composting process (Li et al., 2020; Tran et al., 2015). Furthermore, *Cellulomonas* was recognized as a keystone taxon in the MS and SD groups, likely due to its high efficiency in degrading lignocellulose (Guan et al., 2022). *Cellulomonas* exhibits keystone taxon in MS and SD groups, which may be related to the higher lignocellulose content in millet straw and sawdust. *Cellulomonas* is an aerobic mesophilic bacterium that degrades cellulose during composting, alongside other organisms like Cytophaga. It plays a significant role in the breakdown of cellulose and contributes to the composting process (Alokika and Singh, 2023). Cellulomonas produce cellulase enzymes, which are crucial for the hydrolysis of cellulose. This enzymatic system includes exoglucanase, endoglucanase, and *β*-glucosidase, which work together to convert cellulose into glucose. The activity of these enzymes is essential for the efficient decomposition of cellulose, making Cellulomonas an important player in composting (Zhang et al., 2020).

**Table 2:** Topological features of the microbial network for different bulking agents.

Treatment group	Positive	Negative	Total	Average degree	Graph density	Average clustering coefficient
CS	339	137	476	11.75	0.15	0.721
MS	35	1	36	1.67	0.04	0.425
WS	345	80	425	10.37	0.13	0.726
SD	418	229	647	15.59	0.19	0.711

### 3.4 Microbial community function

**Figure 4:**
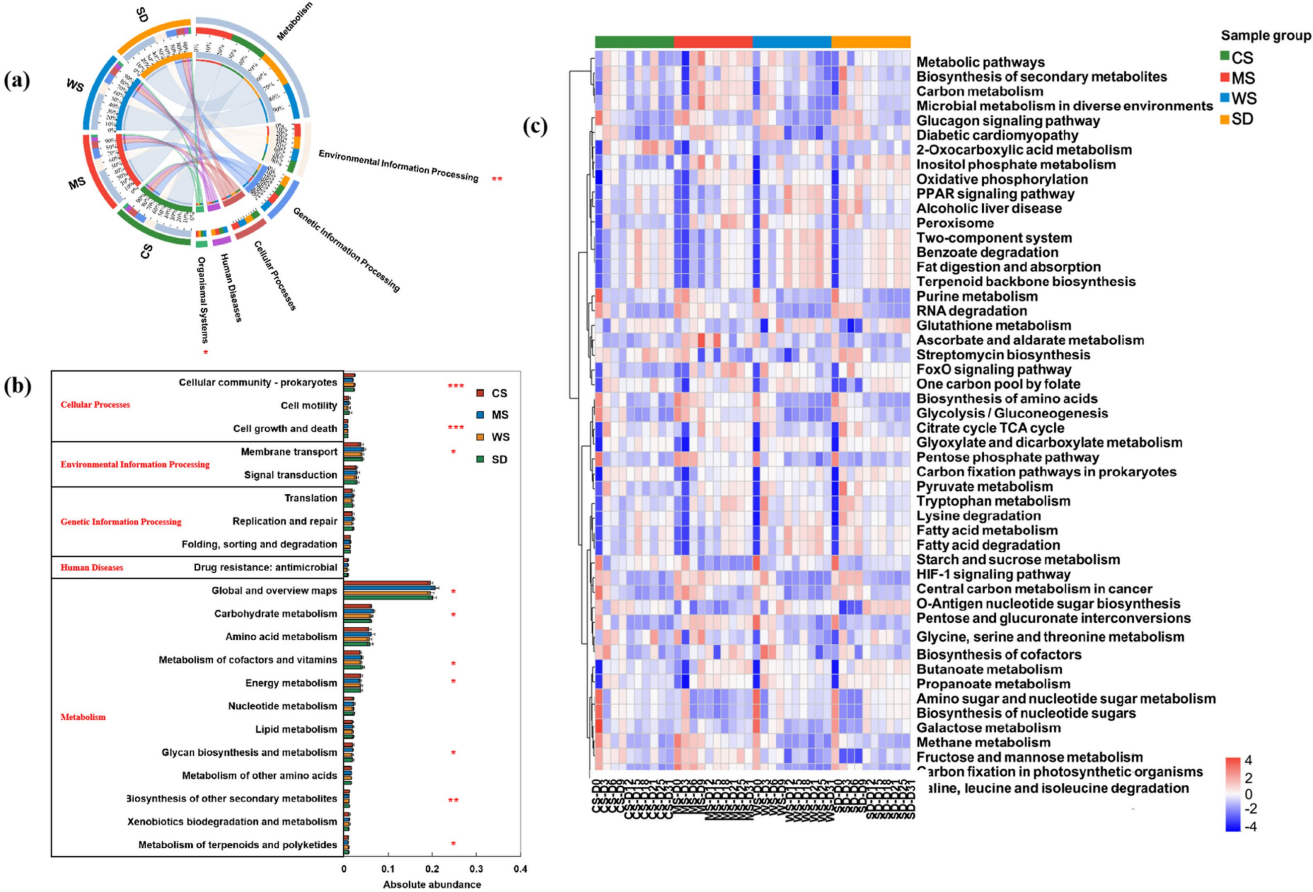
Differences in microbial function of human feces compost with different bulking agents. **(a)** level-1 pathway, **(b)** level-2 pathways with relative abundance greater than 1%, **(c)** top 50 level-3 pathways. The color of the elements in the heatmap is determined by the relative abundance of genes in the corresponding pathway.

To further elucidate changes in microbial community function during composting, we annotated the metagenomic data using the KEGG pathway database. Six metabolic pathways were identified in the level-1 functional group: Metabolism (49.51%–50.12%), Environmental Information Processing (16.17–17.56%), Genetic Information Processing (13.95–14.34%), Cellular Processes (9.62–10.67%), Human Diseases (5.46–5.60%), and Organismal Systems (3.17–3.77%) (Supplementary Table S2). Notably, according to Figure 4a, the abundance of Environmental Information Processing genes was comparable among the CS, WS, and SD groups but was found to be higher in the MS group.

Based on the level-2 pathways, 21 dominant pathways (relative abundance >1%) were identified during human feces composting. As shown in Figure 4b, these include 12 pathways related to Metabolism, one to Human Diseases, three to Genetic Information Processing, two to Environmental Information Processing, and three to Cellular Processes. The primary metabolic pathways were Global and overview maps (26.88–27.39%), Carbohydrate metabolism (7.65–8.33%), and Amino acid metabolism (7.47–7.76%), aligning with previous composting studies (Liang et al., 2020; Wei et al., 2018). Notably, Global and overview maps and Carbohydrate metabolism were significantly elevated in the MS group, highlighting their essential roles in hemicellulose, cellulose, and lignin degradation during aerobic composting (Toledo et al., 2017). During composting, microbial communities primarily drive carbohydrate metabolism linked to lignocellulose degradation and amino acid metabolism associated with nitrogen conversion (Shi et al., 2021). The MS group exhibited notably higher *Bacillus* abundance, a genus pivotal for organic matter and lignocellulose decomposition. This suggests that millet straw may stimulate the proliferation of functional microbes like *Bacillus*, thereby elevating the abundance of carbohydrate metabolism genes (level-2 pathways)—a trend absent in the CS, WS, and SD groups. Notably, millet straw contains relatively low cellulose, hemicellulose, and lignin (Table 1). While high lignocellulose content impedes microbial utilization of cellulose/hemicellulose (Wu et al., 2025; Zhai et al., 2018), our findings imply a potential threshold for lignocellulose content in composting bulking agents: levels below this threshold may enhance decomposition, whereas excess lignocellulose could inhibit it. Conversely, the pathway for Biosynthesis of other secondary metabolites showed the lowest abundance in the MS group. Amino acids, serving as both carbon sources and energy for bacterial metabolism, are known to enhance microbial growth and activity (López-González et al., 2015).

Functional changes during composting were analyzed based on level-3 pathways. As shown in Figure 4c, increased abundances of various third-level metabolic pathways at the composting end relative to the initial stage indicate a shift in microbial activity. Gene abundances in “Metabolic pathways,” “Biosynthesis of secondary metabolites,” and “Microbial metabolism in diverse environments” rose during the thermophilic phase and then declined, suggesting high temperatures reflect elevated metabolic activity. The MS group exhibited significantly lower abundance in “Biosynthesis of secondary metabolites” but higher abundance in “Carbon metabolism” and “Microbial metabolism in diverse environments” than other groups, indicating potentially greater degradation of large organic molecules (Xu et al., 2024). The significant differences in “Carbon metabolism” and “Microbial metabolism in diverse environments” among groups imply that different bulking agents influence microbial utilization of metabolic products, amino acids, and carbohydrates as carbon sources in human feces compost (Li et al., 2023a; Ning et al., 2019).

### 3.5 Association of physicochemical factors with microbial community structure and functional genes

**Figure 5:**
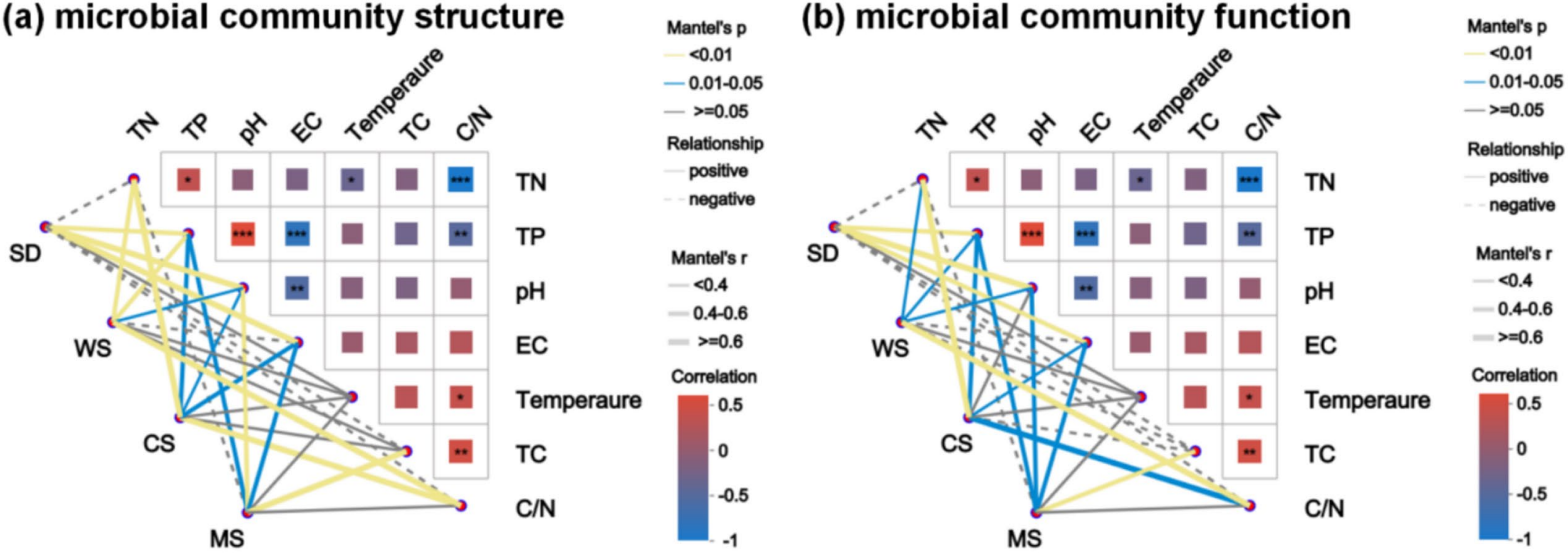
Mantel-test heat map: the lines in the figure represent the correlation between microbial community structure, microbial community function and environmental factors, and the heat map represents the correlation between environmental factors. Line thickness: the correlation between community and environmental factors is drawn with Mantel ‘r (absolute value of r);, relationship: Positive and Negative are the positive and negative correlation between community and environmental factors; Different colors in the heat map represent positive and negative correlations, color depth represents the magnitude of positive and negative correlations, and asterisks in color blocks represent significance, ^*^0.01 < *p* ≤ 0.05, ^**^0.001 < *p* ≤ 0.01, ^***^p ≤ 0.001. **(a)** Microbial community structure, **(b)** Microbial community function.

Pairwise Spearman correlation analysis indicated a positive correlation between TN and TP (r = 0.33, *p* < 0.05), while both were negatively correlated with C/N (r = −0.91, *p* < 0.001 and r = −0.44, *p* < 0.01, respectively). C/N was positively correlated with TC (r = 0.45, *p* < 0.01) and temperature (r = 0.37, *p* < 0.05). EC showed negative correlations with pH (r = −0.5, *p* < 0.01) and TP (r = −0.74, *p* < 0.001). Mantel test analysis was conducted to explore the relationships between physicochemical properties, microbial community structure, and functional attributes across different human feces compost groups. As illustrated in Figure 5, TP and pH emerged as key environmental factors influencing microbial structure and function in compost, aligning with previous findings (Feng and Zhang, 2024). pH suitability governs microbial activity and community succession during composting, serving as a primary factor affecting compost microorganisms (Liu N. et al., 2024). Variations in TN, EC, and C/N have also been linked to microbial community dynamics in human feces compost, highlighting the central role of microorganisms in nitrogen conversion and organic matter degradation during composting (Meng Q. X. et al., 2019; Wang et al., 2019).

### 3.6 Influence of different factors on humification degree of compost

**Figure 6:**
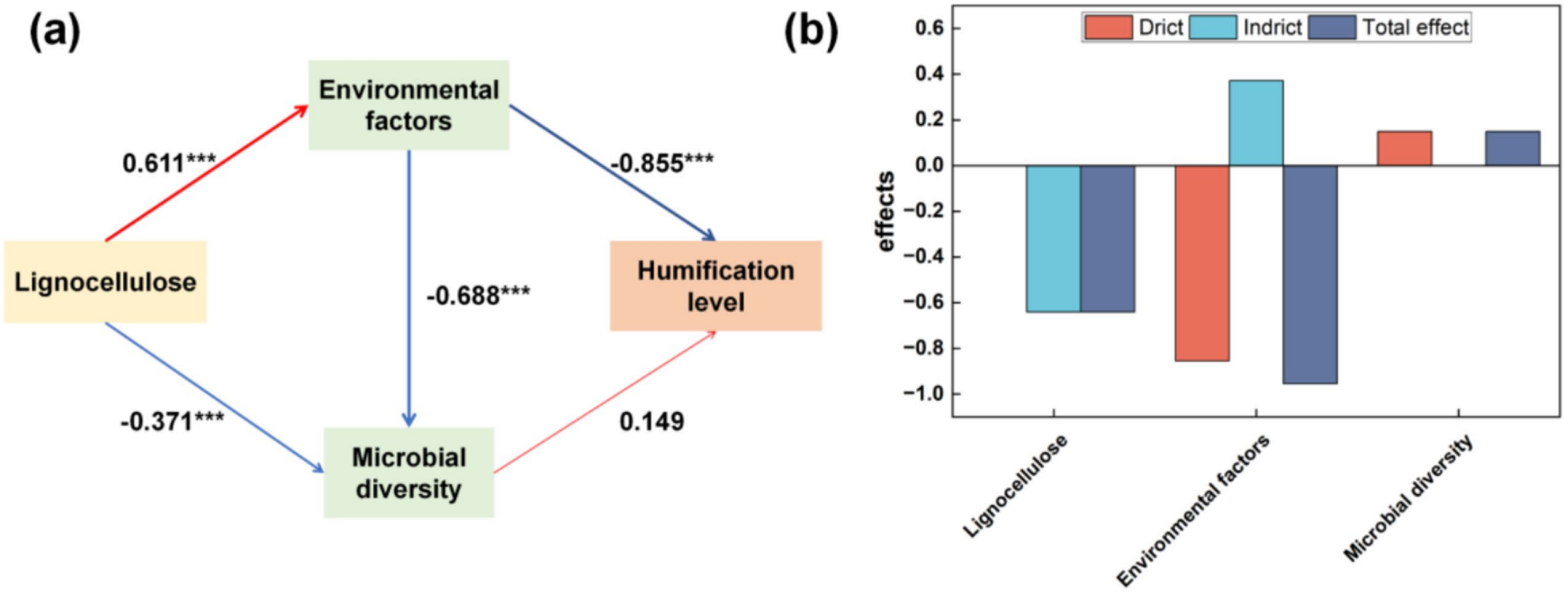
Based on the partial least squares path model, the effects of Lignocellulose content of compost bulking agents, environmental factors, and microbial diversity on the humification degree of compost [**(a)** mutual effects. **(b)** Influence on the degree of humification]. Red indicates a positive path and blue indicates a negative path.

To elucidate the impact of various bulking agents on the composting process, partial least squares regression was employed to evaluate the direct and indirect influences of disparate variables on compost maturation levels. Figure 6 illustrates that humification in human compost is primarily influenced by physicochemical properties, exhibiting a direct effect of −0.855 and a comprehensive effect of −0.955. The physicochemical attributes are directly influenced (0.611) by the lignocellulosic content of the bulking agents. Furthermore, the interaction between lignocellulosic composition and physicochemical characteristics affects microbial diversity, albeit without significant implications for humification. These findings suggest that variations in compost humification are attributable to differences in the lignocellulosic composition of bulking agents. Agroforestry residues characterized by recalcitrant lignocellulosic structures pose considerable challenges to biodegradation during composting, attributed to their slow degradation rates (Guo et al., 2024), with lignocellulose breakdown being a principal bottleneck in composting efficiency (Ma et al., 2024; Wang Q. et al., 2017). The lignocellulose content of the bulk agents added in the four experimental groups is: sawdust > wheat straw > corn straw > millet straw. The microbial diversity of the four experimental groups is: CS > WS > MS > SD. Lignocellulosic components consist of polysaccharides protected by lignin, which hinders enzyme activity. Removing lignin is essential for cellulases and xylanases to access polysaccharide fibers, thereby regulating microbial activities involved in lignocellulose degradation (Asgher et al., 2014). The concentration of lignocellulose derivatives plays a significant role. Microorganisms generally tolerate lower concentrations better, which can even stimulate their activity. However, higher concentrations may inhibit microbial processes (Aboudi et al., 2021). The high content of lignocellulose in sawdust may be one of the reasons for the low microbial diversity in the SD group. Increased secretion of lignocellulose-degrading enzymes promotes microbial growth and metabolism by facilitating the degradation of lignocellulose into glucose, thus regulating microbial activities in fermentation processes (Shishan et al., 2022). Interactions among microorganisms, both competitive and synergistic, are crucial for understanding lignocellulose degradation mechanisms. Specific bacteria enhance cellulose and lignin hydrolysis, thereby regulating microbial activities during composting (Yang et al., 2024). Extracellular fungal enzymes in lignocellulose-rich materials produce growth substrates for bacteria, influencing microbial dynamics. Bacterial activity can modulate fungal enzymatic activity, showcasing the interface interaction mechanisms between these microorganisms (Carneiro et al., 2023). Choosing bulk agents with lower lignocellulose content can improve the effectiveness of human feces composting.

## 4 Conclusion

Human feces can achieve effective humification and maturation when composted with appropriate bulking agents. Among the tested bulking agents—millet straw, sawdust, wheat straw, and corn straw—Corn straw demonstrated superior maturity, higher composting temperatures, shorter thermophilic phases and composting cycles (20d), as well as elevated nutrient levels. The HA/FA of the compost product reached the highest 2.93, the seed germination index attained the highest 112.2%, and the TN concentration achieved the highest 42.87 g/kg. Composting human feces with corn straw also resulted in greater microbial diversity compared to the other agents. Key microbial taxa during the composting process included human commensal flora, lactic acid bacteria, and lignocellulose-degrading bacteria. Under the conditions of C/N = 20 and moisture content of 60%~70%, using corn stalks as bulk agents for human feces composting yields the best results.

The selected bulk agents can improve the quality and efficiency of composting, but continuous research should still be conducted on the environmental impact of human feces composting, including the removal of emitted gases and new pollutants. Still, comprehensive evaluation of agricultural utilization of compost products is also needed. Their effects on the atmospheric environment, as well as the impact of compost products on soil quality, to ensure that human feces composting is environmentally friendly in practice. At the same time, we should continue to explore how to remove new pollutants from human feces compost. And the economic benefits of human feces composting projects should also be evaluated, including cost–benefit analysis, market demand, and competition factors, to ensure their feasibility and sustainability in commercial applications.

## Data Availability

The original contributions presented in the study are publicly available. This data can be found here: https://www.ncbi.nlm.nih.gov/bioproject/, accession number: PRJNA1268696.

## Footnotes

^1^ www.illumina.com

^2^ www.majorbio.com

^3^https://github.com/OpenGene/fastp, version 0.20.0.

^4^https://github.com/voutcn/megahit, version 1.1.2.

^5^https://github.com/hyattpd/Prodigal, version 2.6.3.

^6^http://www.bioinformatics.org/cd-hit/, version 4.6.1.

^7^https://github.com/ShujiaHuang/SOAPaligner, version soap 2.21 release.

^8^http://ab.inf.uni-tuebingen.de/software/diamond/, version 2.0.13.

^9^ http://www.genome.jp/kegg/
