# Supplementary material for: Effect of different bulking agents on the quality, microbial community structure and metabolic functions during human feces composting in foam composting device
Source: Front Microbiol. 2025 Jun 10;16:1556537. doi: 10.3389/fmicb.2025.1556537 (PMC12185541; doi:10.3389/fmicb.2025.1556537)
Supplement: Supplementary file 1 [file Data_Sheet_1.docx]

**Supplementary Information**


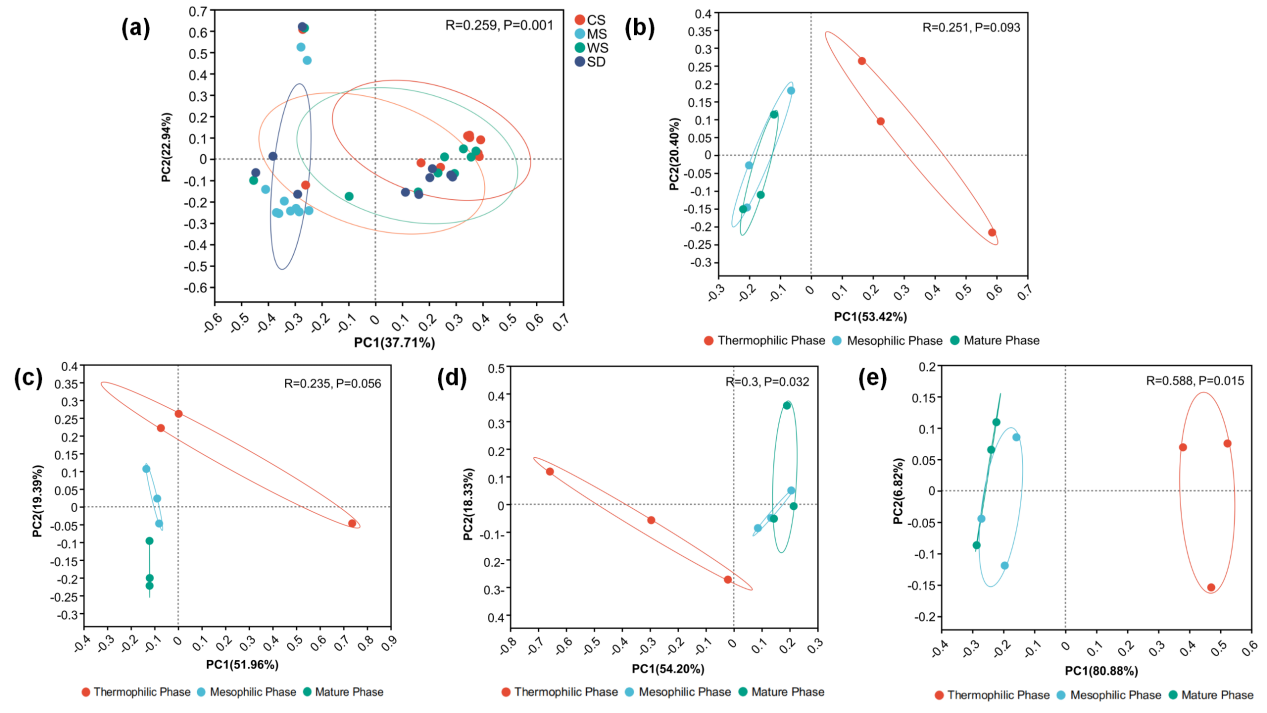


Figure S1 (a) PCoA analysis of microbial community at genus level among different treatment groups, (b),(c),(d) and (e) PCoA analysis of microbial community at genus level among different treatment groups at different composting stages, (b) CS group, (c) MS group, (d) WS group and (e) SD group.


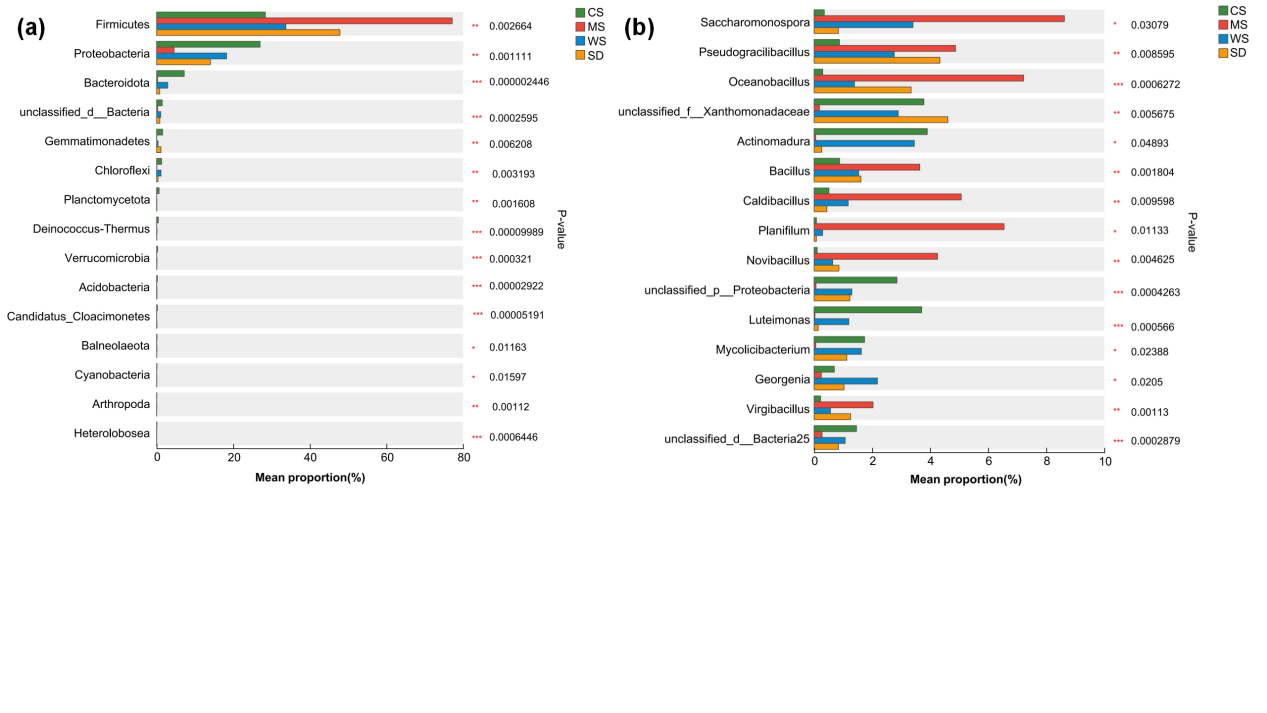


Figure S2 Test of microbial differences between groups (a) phyla level, (b) genus level.

Table S1 Moisture content and C/N ratio of composting raw materials

|  | Moisture Content (%) | C/N |
| --- | --- | --- |
| HF | 81.11 | 12.25 |
| CS | 10.21 | 48.17 |
| MS | 11.05 | 53.81 |
| WS | 10.52 | 60.23 |
| SD | 13.58 | 151.12 |

Table S2 Relative abundance of primary metabolic pathway genes in each group

|  | Metabolism | Environmental Information Processing | Genetic Information Processing | Cellular Processes | Human Diseases | Organismal Systems |
| --- | --- | --- | --- | --- | --- | --- |
| CS | 49.76% | 16.03% | 13.48% | 10.73% | 6.12% | 3.88% |
| MS | 50.21% | 17.24% | 13.58% | 9.53% | 6.08% | 3.36% |
| WS | 49.71% | 16.55% | 13.25% | 10.47% | 6.10% | 3.92% |
| SD | 49.67% | 16.61% | 13.70% | 10.59% | 5.85% | 3.59% |

Table S3 Keystone genera information of different treatment groups

| **group** | **genus** | **degree** | **closeness centrality** | **positive link** | **negatice link** |
| --- | --- | --- | --- | --- | --- |
| CS | g__Enterococcus | 25 | 0.583333333 | 17 | 8 |
|  | g__Erysipelothrix | 25 | 0.644736842 | 13 | 12 |
|  | g__unclassified_o__Myxococcales | 24 | 0.576470588 | 8 | 16 |
|  | g__Peptoniphilus | 23 | 0.569767442 | 15 | 8 |
|  | g__Streptococcus | 23 | 0.604938272 | 12 | 11 |
|  | g__Savagea | 23 | 0.532608696 | 13 | 10 |
|  | g__unclassified_c__Alphaproteobacteria | 23 | 0.576470588 | 9 | 14 |
|  | g__Ligilactobacillus | 22 | 0.521276596 | 14 | 8 |
|  | g__Lapidilactobacillus | 21 | 0.532608696 | 15 | 6 |
|  | g__unclassified_f__Chitinophagaceae | 21 | 0.515789474 | 8 | 13 |
| MS | g__unclassified_o__Eubacteriales | 5 | 0.611111111 | 5 | 0 |
|  | g__Collinsella | 3 | 0.5 | 3 | 0 |
|  | g__Cellulomonas | 3 | 0.8 | 3 | 0 |
|  | g__Lactiplantibacillus | 3 | 0.44 | 2 | 1 |
|  | g__Bifidobacterium | 2 | 0.392857143 | 2 | 0 |
|  | g__Anaerococcus | 2 | 0.323529412 | 2 | 0 |
|  | g__Neobacillus | 2 | 1 | 2 | 0 |
|  | g__unclassified_c__Gammaproteobacteria | 2 | 1 | 2 | 0 |
|  | g__Georgenia | 2 | 0.571428571 | 2 | 0 |
|  | g__unclassified_o__Thermomicrobiales | 2 | 0.571428571 | 2 | 0 |
| WS | g__Enterococcus | 24 | 0.569767442 | 17 | 7 |
|  | g__Actinomadura | 22 | 0.556818182 | 7 | 15 |
|  | g__Amylolactobacillus | 22 | 0.538461538 | 16 | 6 |
|  | g__Loigolactobacillus | 20 | 0.52688172 | 14 | 6 |
|  | g__Mycolicibacterium | 19 | 0.550561798 | 11 | 8 |
|  | g__Lapidilactobacillus | 19 | 0.521276596 | 14 | 5 |
|  | g__Peptoniphilus | 19 | 0.521276596 | 14 | 5 |
|  | g__unclassified_c__Actinomycetia | 19 | 0.532608696 | 5 | 14 |
|  | g__Actinotalea | 19 | 0.563218391 | 10 | 6 |
|  | g__Bacillus | 18 | 0.774193548 | 15 | 3 |
| SD | g__Peptostreptococcus | 36 | 0.383084577 | 20 | 16 |
|  | g__Peptoniphilus | 36 | 0.383084577 | 20 | 16 |
|  | g__unclassified_c__Chloroflexia | 36 | 0.383084577 | 15 | 21 |
|  | g__Georgenia | 35 | 0.379310345 | 14 | 21 |
|  | g__unclassified_o__Thermomicrobiales | 35 | 0.379310345 | 14 | 21 |
|  | g__Eubacterium | 33 | 0.379310345 | 19 | 14 |
|  | g__Erysipelothrix | 32 | 0.371980676 | 17 | 15 |
|  | g__Vagococcus | 31 | 0.371980676 | 19 | 12 |
|  | g__Actinotalea | 31 | 0.371980676 | 15 | 16 |
|  | g__Cellulomonas | 31 | 0.371980676 | 15 | 16 |

Table S4 Temperature changes during the composting process of each composting group (℃)

| Day | 0 | 1 | 2 | 3 | 4 | 5 | 6 | 7 | 8 | 9 | 10 | 11 | 12 | 13 | 14 | 15 | 16 | 17 | 18 | 19 | 20 | 21 | 22 | 23 | 24 | 25 | 26 | 27 | 28 | 29 | 30 | 31 |
| --- | --- | --- | --- | --- | --- | --- | --- | --- | --- | --- | --- | --- | --- | --- | --- | --- | --- | --- | --- | --- | --- | --- | --- | --- | --- | --- | --- | --- | --- | --- | --- | --- |
| CS | 36 | 41.1 | 71.2 | 65.3 | 64.9 | 65.2 | 60.6 | 54.8 | 44.8 | 46.3 | 52.4 | 51.5 | 47.9 | 45.2 | 40.4 | 37.9 | 37.9 | 34.8 | 36.4 | 36.7 | 37.2 | 35.0 | 28.4 | 34.8 | 36.1 | 35.0 | 34.8 | 34.3 | 36.0 | 29.3 | 27.1 | 27.1 |
| MS | 31.8 | 34.5 | 36.4 | 45.9 | 51.9 | 59.5 | 65.7 | 63.7 | 60.3 | 70.7 | 67.3 | 68.7 | 63.8 | 66.6 | 55.1 | 60.9 | 60.9 | 47.8 | 57.5 | 42.7 | 38.7 | 34.2 | 30.0 | 33.9 | 36.1 | 35.6 | 34.5 | 34.2 | 37.1 | 29.9 | 27.9 | 26.6 |
| WS | 36.5 | 38.7 | 65.7 | 67.1 | 64.0 | 66.1 | 62.1 | 60.1 | 46.1 | 49.1 | 54.4 | 47.1 | 44.8 | 49.3 | 45.2 | 41.2 | 41.2 | 35.6 | 38.4 | 35.7 | 37.1 | 35.0 | 28.9 | 35.3 | 36.0 | 34.7 | 34.4 | 34.1 | 36.5 | 30.7 | 27.1 | 26.8 |
| SD | 33.9 | 35.0 | 64.4 | 64.4 | 64.6 | 59.0 | 60.2 | 56.9 | 57.1 | 55.8 | 55.2 | 56.6 | 59.2 | 59.7 | 65.0 | 59.9 | 59.9 | 42.9 | 49.5 | 52.9 | 49.2 | 41.8 | 36.6 | 39.2 | 41.3 | 38.9 | 37.2 | 37.3 | 36.7 | 30.6 | 27.6 | 27.0 |
| ambient temperature | 31.1 | 32 | 34.3 | 35.5 | 38.6 | 32.7 | 35.3 | 28.7 | 32.8 | 39.5 | 40.3 | 39.8 | 40.3 | 33.4 | 36.5 | 36.4 | 26.1 | 35.1 | 38.6 | 32.6 | 36.5 | 34.6 | 29.9 | 39.7 | 39.8 | 36.9 | 37.9 | 38.5 | 37.5 | 31.5 | 28.1 | 27.2 |

Table S5 EC changes during the composting process of each composting group

(μs/cm)

| Day | 0 | 3 | 6 | 9 | 12 | 15 | 18 | 21 | 25 | 31 |
| --- | --- | --- | --- | --- | --- | --- | --- | --- | --- | --- |
| CS | 5.9 | 6.4 | 6.5 | 3.7 | 3.78 | 4.1 | 4.25 | 3.59 | 4.38 | 4.79 |
| MS | 7.6 | 8.4 | 9 | 3.51 | 3.68 | 3.78 | 3.64 | 3.77 | 6.65 | 3.9 |
| WS | 5.1 | 5.6 | 5.8 | 4.83 | 4.37 | 5.3 | 5.1 | 5.4 | 5.3 | 4.04 |
| SD | 5.8 | 6.5 | 6.9 | 2.39 | 2.41 | 2.72 | 2.61 | 2.82 | 3.01 | 3 |

Table S6 pH changes during the composting process of each composting group

| Day | 0 | 3 | 6 | 9 | 12 | 15 | 18 | 21 | 25 | 31 |
| --- | --- | --- | --- | --- | --- | --- | --- | --- | --- | --- |
| CS | 6.84 | 6.9 | 6.84 | 7.24 | 7.1 | 6.9 | 7.06 | 7.17 | 7.13 | 7.05 |
| MS | 7.3 | 7.43 | 7.03 | 7.6 | 7.87 | 7.99 | 8.09 | 8.18 | 8.13 | 8.03 |
| WS | 6.97 | 6.96 | 6.91 | 6.93 | 7.06 | 7.13 | 7.17 | 7.08 | 7.08 | 7.14 |
| SD | 7.25 | 7.41 | 7.12 | 7.4 | 7.49 | 7.53 | 7.44 | 7.53 | 7.51 | 7.55 |

Table S7 TN changes during the composting process of each composting group (g/kg)

| Day | 0 | 3 | 6 | 9 | 12 | 15 | 18 | 21 | 25 | 31 |
| --- | --- | --- | --- | --- | --- | --- | --- | --- | --- | --- |
| CS | 17.83 | 24.45 | 27.99 | 33.06 | 39.12 | 37.98 | 37.21 | 32.32 | 40.70 | 42.87 |
| MS | 24.01 | 26.43 | 25.12 | 24.18 | 27.23 | 24.80 | 23.85 | 30.68 | 13.68 | 27.32 |
| WS | 17.69 | 15.29 | 18.59 | 24.80 | 26.90 | 24.19 | 26.48 | 24.35 | 18.92 | 29.78 |
| SD | 20.95 | 21.72 | 17.79 | 17.43 | 24.35 | 22.44 | 18.38 | 27.91 | 14.07 | 27.44 |

Table S8 TP changes during the composting process of each composting group (g/kg)

| Day | 0 | 3 | 6 | 9 | 12 | 15 | 18 | 21 | 25 | 31 |
| --- | --- | --- | --- | --- | --- | --- | --- | --- | --- | --- |
| CS | 7.82 | 8.84 | 10.97 | 61.51 | 58.35 | 60.48 | 63.73 | 54.10 | 43.21 | 43.55 |
| MS | 9.70 | 10.29 | 11.01 | 53.39 | 58.92 | 81.24 | 62.29 | 74.99 | 52.08 | 46.79 |
| WS | 7.64 | 7.70 | 10.32 | 46.29 | 48.46 | 57.74 | 56.19 | 28.35 | 32.45 | 33.96 |
| SD | 11.04 | 12.08 | 11.04 | 59.44 | 74.06 | 61.20 | 43.91 | 78.24 | 58.56 | 47.16 |

Table S9 HF (g/kg), FA (g/kg), and HA/FA at the end of composting in different composting groups

|  | HA | FA | HA/FA |
| --- | --- | --- | --- |
| CS | 96.2 | 32.8 | 2.9 |
| MS | 53.6 | 48.4 | 1.1 |
| WS | 78 | 44 | 1.8 |
| SD | 39 | 35.9 | 1.1 |
